# Functional mapping of auditory responses to complex sounds under light anesthesia in rhesus macaques

**DOI:** 10.12688/openreseurope.20953.1

**Published:** 2025-09-05

**Authors:** Régis Trapeau, Luc Renaud, Melina Cordeau, Yoan Esposito, Pascal Belin

**Affiliations:** 1 Institut de Neurosciences de la Timone, Aix-Marseille Université, UMR 7289 CNRS, Marseille, Provence-Alpes-Côte d'Azur, 13005, France; 2 Department of Human Evolutionary Biology, Harvard University, Cambridge, Massachusetts, USA; 3 Institute of Language Communication and the Brain, Marseille, Provence-Alpes-Côte d'Azur, 13000, France

**Keywords:** fMRI, auditory, non-human primate, voice, anesthesia

## Abstract

We investigated the auditory responses of anesthetized rhesus macaques to complex natural sounds using functional magnetic resonance imaging (fMRI) and a near-awake anesthesia protocol combining low-dose sevoflurane and dexmedetomidine. Eleven animals were scanned while listening to macaque vocalizations and non-vocal sounds. Robust activation was observed in primary and belt auditory cortices as well as subcortical structures, indicating preserved auditory responsiveness under anesthesia. However, unlike in awake animals, selective responses to vocalizations in anterior temporal voice areas (aTVAs) were absent. Instead, vocalization sensitivity emerged in the ventral premotor cortex, prefrontal cortex, and posterior middle temporal gyrus, regions associated with the dorsal auditory stream and multisensory integration. These findings suggest that while anesthesia preserves basic auditory processing, it modulates higher-order cortical responses involved in voice perception. Anesthetized fMRI thus offers a valuable tool for large-scale studies of primary auditory functions, while it must be approached with caution when studying higher-order auditory functions.

## Plain summary

Understanding how primates process sounds, especially vocalizations, is essential to uncovering the roots of human speech and communication. Typically, this kind of brain research in monkeys is done while the animals are awake, which is challenging and time-consuming. In this study, we used a gentle anesthesia protocol in eleven macaques to see if their brains could still respond to different types of sounds during functional magnetic resonance imaging. We found strong responses in early auditory regions, showing that these parts of the brain still process complex sounds under light anesthesia. However, auditory areas usually involved in processing voices in awake animals did not show the same patterns. Surprisingly, some areas linked to action and multisensory processing were still sensitive to vocalizations. This means that while light anesthesia can help with large-scale brain studies of basic hearing, it may not fully reveal how the brain processes more complex sounds like voices.

## Introduction

Over the past two decades, functional magnetic resonance imaging (fMRI) in rhesus macaques has become a key tool in understanding the neural mechanisms underlying auditory perception, from basic — tonotopy ( Joly *et al*., 2014; Tanji *et al*., 2010), auditory cortical fields mapping ( Petkov *et al*., 2006; Schönwiesner *et al*., 2015), temporal and spectral modulations ( Erb *et al*., 2019) — to higher-level processes — complex sounds ( Kikuchi *et al*., 2014), sound location ( Ortiz-Rios *et al*., 2017), multimodal integration ( Froesel *et al*., 2022; Kayser *et al*., 2005).

Furthermore, fMRI with macaques has contributed to our understanding of the origins of speech by revealing cortical regions that are sensitive to voices and vocalizations in our close relatives ( Bodin *et al*., 2021; Joly *et al*., 2012; Ortiz-Rios *et al*., 2015; Petkov *et al*., 2008), as well as to guide electrophysiological recordings to those regions ( Esposito *et al*., 2025; Giamundo *et al*., 2024; Perrodin *et al*., 2010; Perrodin *et al*., 2011). Such studies have shown that conspecific vocalizations selectively activate higher-level areas of the auditory cortex, particularly in an anterior portion of the temporal gyrus, analogous to the anterior temporal voice area (aTVA) observed in humans ( Aglieri *et al*., 2018; Pernet *et al*., 2015). Furthermore, vocalization-sensitive regions have been reported in areas beyond the temporal gyrus, specifically in the ventral premotor and prefrontal cortices ( Bodin *et al*., 2021; Ortiz-Rios *et al*., 2015).

Auditory fMRI studies in macaques have essentially been performed in awake, behaving animals. Awake fMRI in macaques is particularly valuable because it captures neural responses under naturalistic conditions, in fully engaged, behaviorally active subjects. In addition to the findings related to voice selectivity, awake fMRI has also demonstrated its efficacy in other modalities, particularly in detecting face-selective areas ( Ku *et al*., 2011; Pinsk *et al*., 2009; Tsao *et al*., 2003). However, awake monkey fMRI poses substantial challenges. The animal requires extensive behavioural training to acclimate to the scanner environment, to remain motionless during long sessions of image acquisition, and to perform an auditory task ( Goense *et al*., 2010; Vanduffel & Farivar, 2014). The acquisition of awake fMRI data from as few as two naïve monkeys can easily take over a year. Consequently, the number of animals used in awake fMRI studies is limited, with only two to three animals per study due to feasibility issues. In addition, and despite the behavioral training, inevitable head and body movements occur during acquisition, even under head restraint. This results in large residual motion artifacts that degrade data quality and increase the amount of data that needs to be collected ( Goense *et al*., 2010). As a point of comparison, it has been determined that up to sixty times the amount of data acquired in humans is required to match response reliability in macaque fMRI ( Norman-Haignere *et al*., 2019).

In contrast to awake imaging, anesthetized fMRI protocols in rhesus macaques significantly simplify data acquisition. Anesthesia nearly eradicates head motion, thereby enabling the collection of higher-quality data. Most importantly, anesthesia eliminates the need for long-term behavioral training to ensure that the animal sits still for extended periods in the scanner, a hostile environment for the animal ( Goense *et al*., 2010), and the surgical procedures concomitant with head fixation. These advantages offer the potential for acquiring data on cohort sizes that are nearly equivalent to those of human fMRI studies, and at a significantly reduced time frame when compared to the whole process employed in studies involving only two awake monkeys.

However, anesthetized fMRI comes with its own set of drawbacks. Anesthetic agents used to induce sedation can markedly alter neural activity and neurovascular coupling ( Slupe & Kirsch, 2018; Zhang, 2022), thereby potentially attenuating or even distorting the fMRI BOLD signal that reflects the underlying neuronal response to auditory stimuli ( Guldenmund *et al*., 2017; Uhrig *et al*., 2016). To maximize the outcome of anesthetized fMRI, the anesthesia protocol must be optimized to induce a state that provides immobilization while minimally interfering with the underlying neural processes that support auditory perception. Various combinations of anesthetic agents have been used to achieve this goal. These “near-awake” protocols include: remifentanyl, an opioid analgesic, associated with mivacurium, a neuromuscular-blocking drug ( Ku *et al*., 2011; Logothetis *et al*., 1999; Petkov *et al*., 2006); moderate propofol sedation, with ( Barttfeld *et al*., 2015) or without ( Uhrig *et al*., 2016) muscle blocking agent (cisatracrium); sufentanil or propofol, supplemented with low-dose isoflurane ( Shi *et al*., 2021); the combination of low-dose halogenated ether (isoflurane or sevoflurane) alongside either nitrous oxide ( Obliger-Debouche *et al*., 2025; Sadagopan *et al*., 2015) or dexmedetomidine ( Autio *et al*., 2020), a sedative that has been shown to relatively preserve functional connectivity in humans ( Guldenmund *et al*., 2017).

The objective of the present study was to map the cerebral (subcortical and cortical) response to complex natural sounds, including vocalizations, in a large group of anesthetized macaques. Eleven rhesus macaques were exposed to macaque vocalizations and non-vocal complex sounds during fMRI acquisitions, while under a near-awake anesthesia protocol involving low-dose sevoflurane combined with dexmedetomidine. Our results show robust activation of core and belt auditory cortices, suggesting that much of the fundamental neural circuitry for auditory perception remains active under this anesthesia protocol. However, in contrast with awake fMRI studies, vocalization selectivity was not observed in higher-order, anterior auditory cortices, but was found in the ventral premotor and prefrontal cortex as well as in the posterior middle temporal gyrus. This suggests different involvement of the ventral and dorsal auditory pathways during anesthesia. We conclude that near-awake anesthesia fMRI is appropriate for studying basic auditory functions in a large cohort of subjects, enabling efficient and reproducible mapping of core auditory areas without the extensive training required for awake imaging, while it may obscure the neural mechanisms underlying complex auditory processing, such as the selective response to conspecific vocalizations.

## Methods

### Animals

Eleven rhesus monkeys (Macaca mulatta), 7 females and 4 males, aged between 1 and 17 years (median = 6) and weighing between 1 and 8 kg (median = 5), were scanned. The animals were sourced from the same colony and were selected based on their small head size, which was required to be compatible with the MRI head coil. All experimental procedures were in compliance with the National Institutes of Health’s Guide for the Care and Use of Laboratory Animals and approved by the Ethical board of Institut de Neurosciences de la Timone (ref 2016060618508941).

### Animal preparation and anesthesia

All scans were performed under anesthesia with a protocol inspired by a previous study ( Autio *et al*., 2025). Anesthesia was induced with an intramuscular injection of glycopyrrolate (10 µg/kg), dexmedetomidine (4.5 μg/kg) and ketamine (6 mg/kg), followed by 2% sevoflurane gas administration through tracheal intubation. Two intravenous catheters were implanted into both saphenous veins to provide fluid support for dexmedetomidine and contrast agent delivery separately. Each animal was then secured in the supine position in an MRI testing chair (Rogue Research, Canada) slided feet-first in the MRI scanner. Prior to MRI acquisitions, respiratory control with mechanical ventilation was initiated, as well as continuous delivery of dexmedetomidine (2–4.5 μg/kg/hr). During anatomical scanning, the concentration of sevoflurane gas was lowered to 1–1.5% (depending on the animal), and during functional scanning, it was reduced to 0.5–1.2%. Ferrous oxide contrast agent (monocrystalline iron oxide nanoparticle, MION, 11 mg/kg) was administered at the end of anatomical scanning, some minutes before functional scanning. The animal's physiological characteristics, which includes heart and respiratory rate, capnometry, and oximetry, were monitored during scanning to ensure its health and well-being, as well as to adjust anesthesia to levels close to awakening. A warming blanket was employed to prevent hypothermia.

### Stimuli & audio apparatus

**Figure 1.**
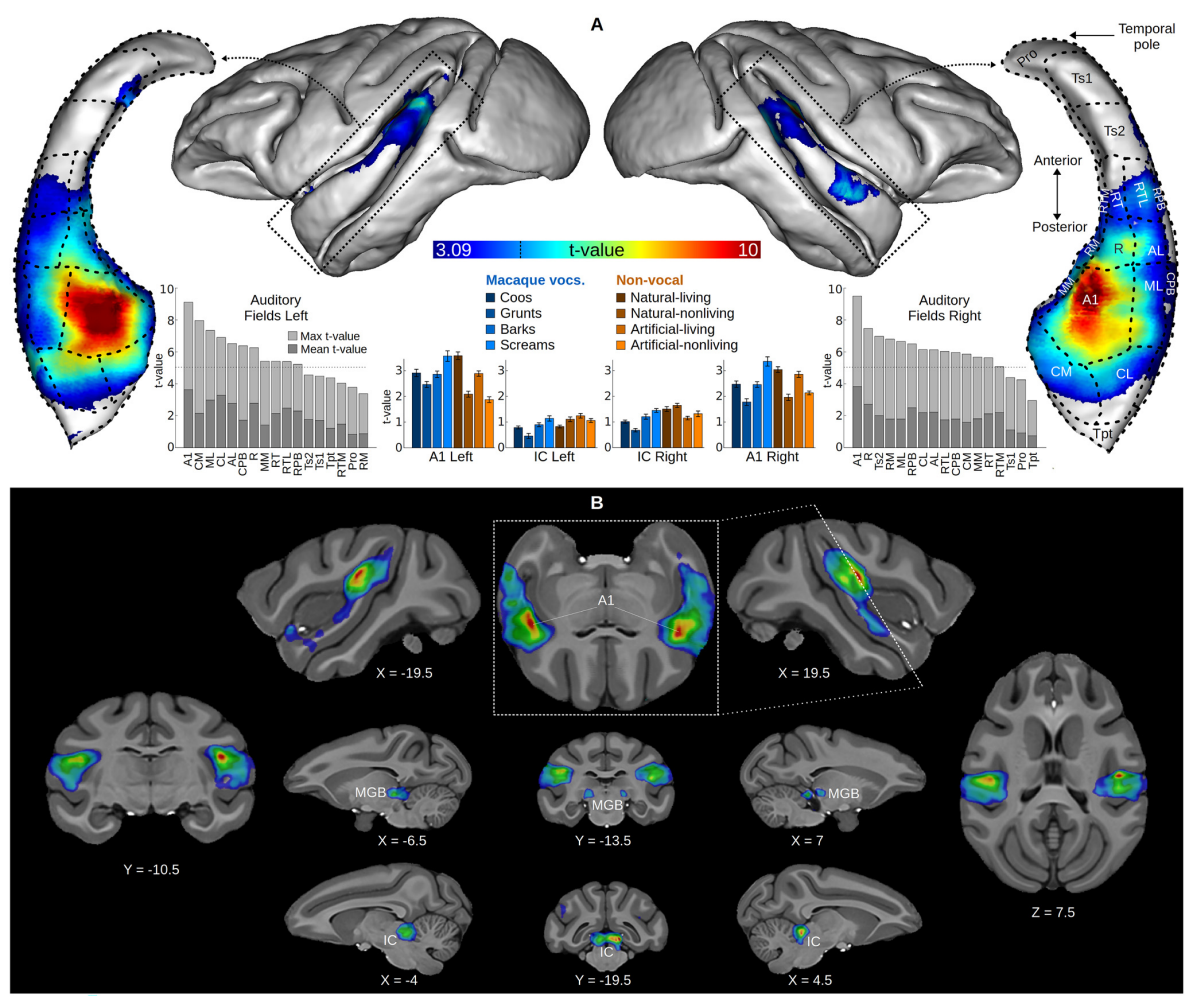
Group-averaged auditory activations. Surface projection and volume map of the “Sound vs. Silence” contrast in a second-level random effects group analysis. For both surface and volume, the contrast map was thresholded at p < 0.05 and corrected for multiple comparisons by cluster size (p < 0.05). The dotted line on the color bar indicates peak threshold corrected for multiple comparisons (t = 4.93) using Gaussian random field theory ( Worsley *et al*., 1996) at p < 0.05. ( **A**) The contrast map is projected onto the surface of the NIMH macaque template (NMT) as well as onto isolated left and right superior temporal gyri viewed from above. Isolated superior temporal gyri include projections of the macaque auditory fields, which were provided by Christopher Petkov’s group ( Petkov *et al*., 2006; Petkov *et al*., 2008). Maximum and mean voxel t-values of each auditory field from the random effects group map are displayed next to the temporal gyri, the horizontal dotted line indicates peak threshold. The panel also shows t-values averaged across individuals (error bars indicate the standard error of the mean) of each subcategory of sound versus silence, extracted from the left and right A1 and IC ROIs (see Methods). ( **B**) The contrast map is overlaid on axial, longitudinal and sagittal slices of the NMT. Top central image is an oblique slice along the superior temporal gyrus. A1: primary auditory cortex; IC: inferior colliculus; MGB: medial geniculate body.

Sound stimuli were a subset of the open access PrimaVoice audio stimulus set ( Bodin *et al*., 2025) and comprised two main categories: macaque vocalizations and non-vocal sounds. Each category contained 24 stimuli, for a total of 48 sound stimuli. Each main category was divided into 4 subcategories of 6 stimuli, forming 8 subcategories in total (see Figure 1). Macaque vocalizations were provided by Marc Hauser ( Hauser, 1991) and included both positive (coos, n = 6, grunts, n = 6) and negative (barks, n = 6, screams, n = 6) calls. Non-vocal sounds included both natural (living, n = 6, non-living, n = 6) and artificial sounds (human actions, n = 6, or not, n = 6) from previous studies from our group ( Belin *et al*., 2000; Capilla *et al*., 2013) or kindly provided by Christopher Petkov ( Petkov *et al*., 2008) and Elia Formisano ( Moerel *et al*., 2012). Stimuli were adjusted in duration, resampled at 48828 Hz and normalized by root mean square amplitude. Finally, a 10-ms cosine ramp was applied to the onset and offset of all stimuli. During experiments, stimuli were digitally processed by a TDT System 3 RM1 ( Tucker Davis Technologies, Alachua, FL, USA) and delivered via three MRI-compatible piezoelectric speakers ( Sonitron, Sint-Niklaas, Belgium). The sound was essentially delivered by bone conduction. Two smaller speakers (frequency response ranging from 1 kHz to 20 kHz) were placed on or near the animal's ears inside the antenna and gently pressed against its skull. A third, larger speaker (frequency response ranging from 300 Hz to 20 kHz), was placed against the dorsal part of the animal’s head through the antenna’s hole designed to house a head post.

### MRI data acquisition

Images were acquired at the MRI Center of the Institut de Neurosciences de la Timone (Marseille, France) using a 3-Tesla PRISMA scanner (Siemens, Erlangen, Germany) with a 24-channels macaque head coil (Rogue Research, Canada) ( Autio *et al*., 2025). High-resolution T1 & T2-weighted anatomical volumes were first acquired for coregistration and normalization purposes (T1w: MPRAGE sequence, TR = 2.2 s, TE = 2.23 ms, TI = 900 ms flip angle: 8°, matrix size = 224 × 256 × 256, resolution 0.5 × 0.5 x 0.5 mm ^3^, bandwidth 270 Hz/Pixel, GRAPPA = 2, sagittal orientation; T2w: SPACE sequence, TR = 3.2 s, TE = 562 ms, matrix size = 224 × 256 × 256, resolution 0.5 × 0.5 x 0.5 mm ^3^, bandwidth 723 Hz/Pixel, turbo factor 314, echo train length 1234 ms, GRAPPA = 2, sagittal orientation).

Fieldmaps B0 field-maps were estimated using a pair of spin-echo EPI images with one opposite phase encoding directions ( Andersson *et al*., 2003) (LR and RL directions, 1.5 mm isotropic, TE 46.2 ms, TR 7.06 s). The spin-echo EPI scans were matched with geometry and distortion properties of fMRI acquisitions (FOV 96 × 96 mm, matrix 64 × 64, number of slices 39, phase partial Fourier 65%, bandwidth 1240 Hz/pixel, echo spacing = 0.93 ms, GRAPPA = 2, fat suppression, flip-angle = 90°, averages 3).

Functional volumes were acquired with the same coil using multiband EPI sequences obtained from the Center for Magnetic Resonance Research (CMRR, University of Minnesota), version R016a (multiband acceleration factor = 3, TA = 646 ms, TE = 16.6 ms, matrix size = 64 × 64 × 39, resolution 1.5 x 1.5 x 1.5 mm ^3^, flip angle = 50°, multiband LeakBlock kernel, GRAPPA = 2 with EPI calibration scan with 18 reference lines, bandwidth = 1240 Hz/Pixel, echo spacing = 0.93 ms, phase partial Fourier = 75%, fat suppression).

### Functional imaging procedure

To avoid interference from the scanner noise during stimulus presentation, clustered sparse sampling of functional acquisitions was employed ( Schmidt *et al*., 2008; Trapeau *et al*., 2024; Zaehle *et al*., 2007). Clusters of seven fMRI volumes (4.6 s) were acquired after a 3.5 s acquisition-free period, which allowed stimuli to be presented when the scanner was silent. During each 7-minute functional run, 32 blocks of stimuli were presented (16 blocks of each of the two main categories), interleaved with 16 silent blocks. Each block contained six stimuli (ISI = 50 ms) belonging to one of the eight subcategories. The time interval between the end of an acquisition cluster and the start of a block of stimuli was randomly jittered from 100 ms to 250 ms. A total of 188 runs were performed among the 11 subjects (one session per subject except for M1 who underwent six sessions; median of 12 runs per subject).

### fMRI data preprocessing

Brain extraction and tissue segmentation was performed on the structural scans using a custom-made pipeline which included: bias field correction using the product of the T1w and T2w images ( Glasser *et al*., 2013); denoising (spatially adaptive nonlocal means, SPM); first brain extraction ( FSL); registration of the NIMH Macaque Template v1.2 ( Seidlitz *et al*., 2018) to the anatomical scan, in order to provide priors to SPM’s old_segment algorithm; second brain extraction from the segmented tissues. Transformation matrices between anatomical and functional data were computed using non-linear registration (SyN, ANTS). Additionally, white matter and cerebrospinal fluid masks were eroded using a 3 mm cubic kernel in order to provide a mask that contains no region of interest to a principal component analysis of the functional data (see *fMRI data analysis*). Transformation matrices between functional, anatomical and template volumes were computed using non-linear registration (SyN, ANTS). These transformation matrices were used to register the segmented tissues to the functional data as well as to register the functional results to individual high-resolution anatomical scans and to the template.

Preprocessing of the functional data included motion correction, spatial distortion reduction using field maps, inter-runs registration and spatial smoothing. For each subject, every functional volume was realigned and unwarped (FSL) to a reference volume that was spatially the closest to the average of all runs. Spatial smoothing (SPM) was done with a full-width half-maximum 3-dimensional Gaussian kernel that was twice the size of the functional voxels (i.e., 3 mm).

### fMRI data analysis

For each subject, general linear model (GLM) estimates of responses to all sounds versus silence (“Sound vs. Silence” contrast) and to macaque vocalizations against non-vocal sounds (“Macaque vs. Non-Vocal” contrast) were computed with fMRISTAT ( Worsley *et al*., 2002) using MION-based response function, manually designed to have a reversed sign, a long tail and no undershoot, similar to previous descriptions ( Leite *et al*., 2002). The GLM included covariates of no interest: the first principal components of two principal component analyses performed on eroded masks of the functional voxels identified as containing white matter and cerebrospinal fluid. The number principal components included as covariates of no interest was chosen to optimize the “Sound vs. Silence” contrast in each subject (6 for white matter, 4 for cerebrospinal fluid, on average).

Following this first level-analysis, fixed and random effects group maps were computed for both contrasts, using the template registered data from all subjects and all runs. Additionally, the group map for the “Macaque vs. Non-Vocal” contrast was computed on a selection of runs that exhibited a positive whole-brain influence on this contrast. The contribution of each run to the contrast was assessed on a per-subject basis using a jackknife procedure. This procedure systematically excluded one run from the dataset of each subject and returned the maximum t-value elicited by the remaining runs. Then, the resulting t-values of a subject were compared with the maximum t-value elicited by all runs of this subject. A lower maximum t-value after run removal meant that the run had a positive contribution to the contrast and vice versa. Note that no topographical constraints were imposed on where these maximum t-values should be, as they were computed using the whole-brain t-maps. Only the runs that showed a positive contribution to the “Macaque vs. Non-Vocal” contrast of each subject were kept, leaving 72 runs in total (median of 6 runs per subject).

T-values of each subcategory were extracted for each subject in several bilateral regions of interest (ROI): the primary auditory cortex (A1), which was defined, for each subject, as the intersection of the A1 auditory field identified by ( Petkov *et al*., 2006) and the “Sound vs. Silence” contrast of the subject; the inferior colliculus (IC), based on peak activations in this anatomically defined region from the random effects group map of the “Sound vs. Silence” contrast; the posterior middle temporal gyrus (pMTG) and the rostral portion of the ventral premotor cortex (F5), both based on peak activations in this region from the fixed effects group map of the “Macaque vs. Non-Vocal” contrast; and the anterior temporal voice area (aTVA) that was previously observed in three macaques during an awake fMRI study by our group ( Bodin *et al*., 2021). Each ROI size was 19 voxels in the functional space and a diameter of 4.5 mm.

## Results

### Auditory activations

Second-level random effects group analysis revealed strong activations in the primary auditory cortex (A1) and nuclei of the ascending auditory pathway (inferior colliculus, IC; medial geniculate body, MGB) in response to all sounds (“Sound vs. Silence” contrast, Figure 1). Cortical activation extended to the rostral field of the core auditory cortex (R), but also to belt areas (CM, CL, ML, AL, RTL, MM, RM, RTM, Figure 1A). The parabelt and higher auditory fields were marginally activated, except for the right rostral parabelt (RPB) and the temporalis superior 2 (Ts2), which showed significant activation in some voxels.

At the individual level, all subjects had significant auditory responsive clusters in IC and in the auditory cortex ( Table 1). ROI t-value extraction showed that all subcategories of sounds elicited positive auditory activations in both IC and A1. ( Figure 1A).

**Table 1.** Individual activations in IC and AC. Peak t-value and number of significant voxels at cluster threshold (t = 3.09) in the inferior colliculi (IC) and auditory cortices (AC) of each subject.

Subject	IC peak t-value	IC extent (voxels)	AC peak t-value	AC extent (voxels)
M1	12.87	105	8.04	271
M2	5.22	27	4.2	23
M3	5.14	20	6.78	110
M4	4.07	15	6.06	257
M5	4.43	13	8.45	344
M6	7.61	40	11.26	367
M7	4.69	13	9.91	361
M8	6.79	45	3.6	11
M9	5.69	19	8.39	278
M10	6.92	32	8.34	235
M11	6.74	33	8.2	198

### Vocalization-specific activations

**Figure 2.**
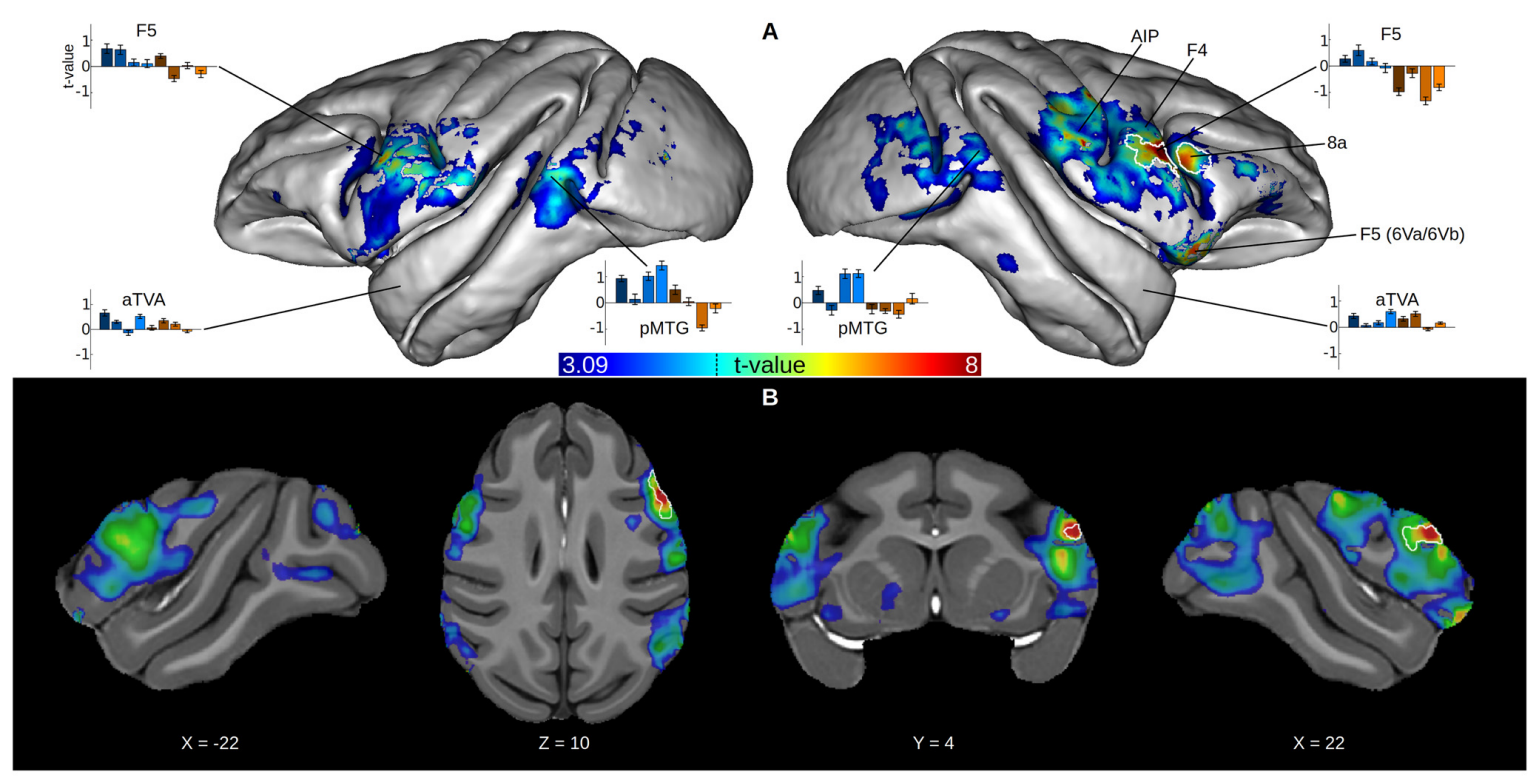
Group-averaged activations to macaque vocalizations. Surface projection and volume map of the “Macaque vs. Non Vocal” contrast in a second-level fixed effects group analysis, computed on a selection of runs that had a positive whole-brain influence on this contrast (see Methods). The white outlines indicate significant areas (p < 0.05, cluster size corrected) of the same analysis computed on all runs. For both surface and volume, the contrast map was thresholded at p < 0.05 and corrected for multiple comparisons by cluster size (p < 0.05). The dotted line on the color bar indicates peak threshold corrected for multiple comparisons using Gaussian random field theory ( Worsley *et al*., 1996) at p < 0.05. ( **A**) The contrast map is projected on the surface of the NMT. The panel also shows t-values averaged across individuals (error bars indicate the standard error of the mean) of each subcategory of sound versus silence, extracted from the several ROIs (see Methods). ( **B**) The contrast map is overlaid on axial, longitudinal and sagittal slices of the NMT. F5: rostral ventral premotor cortex; F4: caudal ventral premotor cortex; AIP: anterior intraparietal area; pMTG: posterior middle temporal gyrus; aTVA: anterior temporal voice area.

We could not reproduce our previous findings of voice-selective areas in the anterior temporal lobe (aTVA) of awake macaques ( Bodin *et al*., 2021) in a second-level, random effects group analysis computed on all runs. However, a fixed effects analysis revealed activations in the rostral ventral premotor cortex (F5) and area 8a of the right hemisphere (white outline in Figure 2).

Next, we conducted the same analyses on a selection of runs that had a positive whole-brain contribution to the contrast (see fMRI data analysis). The random effects group map computed from this selection of 72 runs was very similar to the fixed effects group map computed from all 188 runs, showing activations in areas F5 and 8a in the right hemisphere. The fixed effects analysis on the selection of runs revealed much broader activations: F5 bilaterally, right anterior intraparietal area (AIP) and caudal ventral premotor cortex (F4), right 6Va/6Vb, posterior part of the middle temporal gyrus (pMTG) bilaterally.

ROI t-value extraction showed showed that auditory activations for each subcategory of sounds in F5 and pMTG were mainly positive for macaque vocalizations and negative for non-vocal sounds ( Figure 2A). This difference between vocalizations and non-vocal sounds was not observed in the awake macaques aTVA, where auditory activations were mainly positive for all subcategories.

## Discussion

This study aimed to map the auditory response to complex natural sounds in a large group of rhesus macaques under a near-awake anesthesia protocol involving low-dose sevoflurane and dexmedetomidine. Using a sizable sample of eleven macaques, which approaches the median of twelve subjects employed in human fMRI studies ( Szucs & Ioannidis, 2020), we have obtained the first random-effects group map of auditory activation in this species. The results revealed robust responses in the core and belt areas of the auditory cortex, as well as in subcortical structures, including the midbrain (inferior colliculi) and thalamus (medial geniculate body). This suggests that essential neural circuitry supporting auditory perception remains functional under this anesthesia regimen. These findings demonstrate the efficacy of anesthetized macaque fMRI in facilitating efficient and high-quality mapping of subcortical and core auditory regions across larger cohorts, thereby circumventing the logistical challenges associated with awake imaging.

Vocalization-specific activations revealed a different picture. To our disappointment, our results revealed a conspicuous absence of vocalization selectivity in higher-order, anterior auditory cortices. This result diverges from previous findings of our group, which highlighted the crucial involvement of the aTVA in processing the same set of stimuli ( Bodin *et al*., 2021), as well as from earlier awake macaque studies ( Ortiz-Rios *et al*., 2015; Petkov *et al*., 2008). This outcome was somewhat unexpected, given that aTVA activation had been previously reported even in anesthetized macaques ( Petkov *et al*., 2008). Nevertheless, a critical distinction exists between this study and ours with respect to the pharmaceutical agents utilized for immobilizing the animals during experimentation. In their study, Petkov and colleagues used remifentanyl and mivacurium, which is the combination of an analgesic with a neuromuscular-blocking drug. However, the absence of hypnotic agents, even at sedation levels, in this cocktail suggests that the animals remained conscious during the experiment. Conversely, we opted for a general anesthesia protocol, involving a sedative agent combined with a hypnotic agent at low dose, with meticulous physiologic monitoring and adjustment of drug levels throughout the experiment to bring the animal close to awakening. Our protocol, involving sevoflurane and dexmedetomidine, prioritized animal well-being and minimized the risk of a non pleasant mnesic experience, though possibly at the cost of reduced activation in anterior auditory regions. The inclusion of each animal in a singular session, in which some completed less than ten trials, might have also been suboptimal for detecting vocalization sensibility in higher-order auditory cortices.

Despite the absence of observed vocalization selectivity in the anterior temporal gyrus, our findings revealed the presence of voice-selective regions in the ventral premotor (F5) and prefrontal cortex (8a). The involvement of these regions in vocalization processing has been previously observed in some awake macaque fMRI studies ( Bodin *et al*., 2021; Ortiz-Rios *et al*., 2015) and they have been identified as part of the dorsal auditory pathway ( Arnott & Alain, 2011; Petrides & Pandya, 2009), which connects the caudal parabelt auditory regions to the ventral inferior parietal cortex ( Lewis & Van Essen, 2000), and up to the frontal eye fields ( Hackett *et al*., 1999; Romanski *et al*., 1999). These findings suggest that under our anesthesia regimen, the dorsal auditory stream may be preferentially engaged, speculatively as a resilient backbone for action planning, over the ventral pathway, which is more dedicated to the identification of auditory objects ( Kuśmierek *et al*., 2012). More surprisingly, we found additional bilateral vocalization sensitivity in the posterior part of the middle temporal gyrus, a region previously identified as a site of audio-visual integration in humans ( Beauchamp *et al*., 2004; Callan *et al*., 2004; Xu *et al*., 2009), macaques ( Froesel *et al*., 2022) and marmosets ( Dureux *et al*., 2024), but, to our knowledge, not reported in auditory only experiments.

In conclusion, while awake fMRI remains indispensable for probing the full complexity of the cortical processing of natural sounds such as conspecific vocalizations, our findings demonstrate that anesthetized fMRI is a powerful and efficient alternative for large-scale mapping of basic auditory function in non-human primates. Our results confirm that primary auditory pathways remain responsive under near-awake anesthesia. However, they also reveal that anesthesia, even at low levels, induces a dampening of higher-order, anterior auditory cortices, where voice-selective responses were previously identified with awake fMRI studies. Nevertheless, vocalization selectivity was observed in the ventral premotor and prefrontal cortex as well as in the posterior middle temporal gyrus, which suggests a different involvement of the ventral and dorsal auditory pathways during anesthesia. These regions have received relatively little attention in the context of vocalization processing, whether in fMRI or electrophysiological studies, where the focus has traditionally been on anterior temporal areas. In light of their pronounced involvement during anesthesia, future research endeavors should consider conducting a more thorough investigation of their specific role in the auditory processing of vocal signals.

## Ethics and consent

All experimental procedures were in compliance with the National Institutes of Health’s Guide for the Care and Use of Laboratory Animals and approved by the Ethical board of Institut de Neurosciences de la Timone (ref 2016060618508941). Efforts were made to ameliorate the suffering of animals using endpoints, environmental enrichment, and careful monitoring of health and well-being.

## Data Availability

Zenodo: Anesthetized Macaque fMRI data - Vocalizations and Non-Vocal Sounds, https://doi.org/10.5281/zenodo.16941258, ( Trapeau & Belin, 2025) Data are available under the terms of the Creative Commons Attribution 4.0 International Zenodo: Audio stimuli - PrimaVoice: A Cross-Species Stimulus Set to Study Voice Processing in Primates, https://doi.org/10.5281/zenodo.15297317, ( Bodin *et al*., 2025) Data are available under the terms of the Creative Commons Attribution 4.0 International

## Software availability statement

Source code available from: https://github.com/mdobraz/PrimaVoice-Toolbox

Archived software available from: https://doi.org/10.5281/zenodo.5075674, ( Trapeau & Belin, 2021)

License: the PrimaVoice Toolbox is licensed under the MIT License.

SPM and fMRISTAT are compatible with GNU Octave which is licensed under the GNU General Public License v3.0.

## Reporting guidelines

We used the ARRIVE checklist when writing our report ( Percie Du Sert *et al*., 2020).

The completed ARRIVE checklist is contained in the underlying data Zenodo record: https://doi.org/10.5281/zenodo.16941258, completed_ARRIVE_checklist.pdf, ( Trapeau & Belin, 2025)
