# Supplementary material for: Functional mapping of auditory responses to complex sounds under light anesthesia in rhesus macaques
Source: Open Res Eur. 2025 Sep 5;5:273. [Version 1] doi: 10.12688/openreseurope.20953.1 (PMC12547387; doi:10.12688/openreseurope.20953.1)
Supplement: Peer review history

# Reviewer report: Reviewer response for version 1

**DOI:** 10.21956/openreseurope.22669.r59877

**Published:** 2025-10-22

**Authors:** Alix Trouillet

**Affiliations:** 1 ORL, Centre Hospitalier Universitaire Vaudois, Lausanne, Vaud, Switzerland

The team looked at the auditory responses to complex natural sound ( vocalization and non-vocal sounds) using fMRI in anesthetized macaques (n=11).  

 They show that response is mostly preserved under anesthesia (near-awake protocol) in primary and belt areas. However, they were unable to reproduce previous findings that showed activation of the aTVA in awake macaques.  

 Instead, they found that voice activation was found in regions associated with the dorsal auditory stream and multisensory integration. 

 The paper is clearly written. The study design is appropriate, the sample size is good (n=11 macaques) and the experiment seems to be replicable. 

 The conclusions are mainly descriptive. It consists of depicting the impact of light anesthesia on auditory brain activity. 

 The conclusions are not completely new in the sense that it was already known that essential neural circuitry supporting auditory perception remained functional under anesthesia in macaques. However, they show that under this regimen unexpected regions are activated by vocal stimuli (ventral premotor and prefrontal cortex) while the anterior temporal voice area is not.  

 The authors conclude that fMRI under near-awake anesthesia can still be used to study basic auditory function in non-human primates but could be limiting when studying the processing of more complex sounds like voices.  

 While interesting, I think it is important to be very cautious when studying brain circuitry under anesthesia. Sedation protocols vary a lot depending on countries, institution and experimenters. It is known that they have a big impact on brain function; their effects are highly variable between species and between individuals making it very difficult to draw and extrapolate clear conclusions.

Is the study design appropriate and does the work have academic merit?

Yes

Is the work clearly and accurately presented and does it cite the current literature?

Yes

If applicable, is the statistical analysis and its interpretation appropriate?

Not applicable

Are all the source data underlying the results available to ensure full reproducibility?

Yes

Are the conclusions drawn adequately supported by the results?

Yes

Are sufficient details of methods and analysis provided to allow replication by others?

Yes

Reviewer Expertise:

I have an expertise in NHP Auditory neuroscience  but more at the electrophysiological level. Extensive experience in comparing awake and anesthesized signals.  However, I am unable to assess the accuracy of fMRI analysis.

I confirm that I have read this submission and believe that I have an appropriate level of expertise to confirm that it is of an acceptable scientific standard.

# Reviewer report: Reviewer response for version 1

**DOI:** 10.21956/openreseurope.22669.r59881

**Published:** 2025-09-27

**Authors:** Kirill V Nourski, Emily Dappen

**Affiliations:** 1 The University of Iowa, Iowa, USA; 2 The University of Iowa Department of Neurosurgery, Iowa City, Iowa, USA

This is an interesting study on the important topics of non-human primate neuroimaging in auditory research and effects of anesthesia on cortical auditory processing. The authors present results obtained in lightly anesthetized macaques and compare them with their previous work obtained in awake animals (Bodin et al., 2021, *Curr Biol. *2021; **31**(21): 4839–4844.e4).

 Major comments:

- Introduction: there is a lot of information on why light anesthesia with fMRI is useful, and the pros and cons of both awake and anesthesia fMRI. However, the Introduction does not provide much information on why the authors are examining speech and auditory processing, why it is beneficial to study, and what it can tell us/how it is translatable to human work. Much of the discussion of prior fMRI studies would probably be better suited for the Discussion section. The authors could discuss the limitations of anesthesia fMRI but then discuss why it is still better (in this case) than awake fMRI to answer the proposed questions.
- Introduction: the hypotheses are not clearly stated.
- Methods: It is not clear how exactly was “light anesthesia” defined and confirmed during the experiments.
- Methods: As the focus of the paper is the comparison between new results obtained under light anesthesia and previously published results (Bodin et al., 2021), it should be explicitly stated that, aside from anesthesia, experimental procedures were identical between the two studies.
- Discussion: the underlying cause(s) of the differences between the present and previously published results (Bodin et al., 2021) should be presented, and alternative explanations should be considered. Is it a difference in arousal/consciousness state? Is it the effect of the drug? Are there any methodological differences between the two studies that could contribute to the observed difference in results?

Other specific comments:

 Abstract:

 “Instead, vocalization sensitivity emerged in… posterior middle temporal gyrus, regions associated with the dorsal auditory stream and multisensory integration”. Posterior MTG is typically considered part of the ventral rather than dorsal stream (e.g., Hickok & Poeppel, 2015, Handb Clin Neurol 129:149-160).

 Keywords:

 “auditory” and “anesthesia” are used verbatim in the title and thus are redundant as keywords.

 Methods

 Pg4, P3: The description of auditory stimuli used is insufficient given the scope of the study. It’s unclear what the “living” and “non-living” stimuli were. It is states that the stimuli were “adjusted for duration”, but the actual duration is not specified. Please consider providing comprehensive details of the stimuli used as a summary table and/or examples of stimulus waveforms or spectrograms in Supplementary materials.

 Figure 1: Referencing figure 1 in the methods was a bit confusing. It might be worth separating out figure 1 into two different figures, one as a methods figure describing the different stimuli and the second with the results.

 Pg5, P1: Please spell out ISI. Also please explain why an inter-stimulus-interval of 50 ms was used. Is this typical for auditory paradigms in rhesus monkeys?

 Results:

 Pg6, P8: “We could not reproduce our previous findings of voice-selective areas in the anterior temporal lobe (aTVA) of awake macaques ( Bodin *et al*., 2021) in a second-level, random effects group analysis computed on all runs.” As is, adding this sentence adds confusion – perhaps rephrasing to just highlight the result then discussing how this diverges from previous findings in the discussion (as the authors have done) would improve clarity.

 Pg8, P2: “This difference between vocalizations and non-vocal sounds was not observed in the awake macaques aTVA, where auditory activations were mainly positive for all subcategories.” Please cite Bodin et al (2021) here if that’s the awake study that is referred to in this sentence.

 Figure 2: Please make the bar plots larger – it is hard to see the error bars and what color each bar is. Please also include a key for what the bar colors mean.

Is the study design appropriate and does the work have academic merit?

Yes

Is the work clearly and accurately presented and does it cite the current literature?

Yes

If applicable, is the statistical analysis and its interpretation appropriate?

Yes

Are all the source data underlying the results available to ensure full reproducibility?

No

Are the conclusions drawn adequately supported by the results?

Partly

Are sufficient details of methods and analysis provided to allow replication by others?

No

Reviewer Expertise:

Auditory neurophysiology, consciousness neuroscience.

We confirm that we have read this submission and believe that we have an appropriate level of expertise to confirm that it is of an acceptable scientific standard, however we have significant reservations, as outlined above.

# Reviewer report: Reviewer response for version 1

**DOI:** 10.21956/openreseurope.22669.r59878

**Published:** 2025-09-26

**Authors:** Mathilda Froesel

**Affiliations:** 1 Neurosciences, KU Leuven Department of Neurosciences (Ringgold ID: 213965), Leuven, Flanders, Belgium; 2 Institut des sciences cognitives Marc Jeannerod, Bron, Auvergne-Rhône-Alpes, France

This study addresses the important question of the neural basis of auditory perception in macaques, utilizing a "near-awake" anesthesia protocol. The authors should be commended for collecting a valuable dataset from a large cohort of eleven subjects, which is a significant effort and a precious resource for the community. The goal of mapping auditory responses and comparing them to findings from awake-animal studies is highly relevant and of great interest. The paper has the potential to make a significant contribution to our understanding of the effects of anesthesia on auditory processing. However, several major methodological concerns must be addressed to ensure the validity and robustness of the conclusions, particularly those regarding vocalization selectivity. 

 Major concerns:

 1) Circular Analysis in Run Selection My most significant concern relates to the procedure for selecting runs for the vocalizations-specific activation analysis. Selecting runs (72 out of 188, meaning over 60% of the data was discarded) based on their "positive contribution" to the contrast of interest is a form of circular analysis ("double-dipping"). This method invalidates the statistical inference, artificially inflates the results, and prevents reliable conclusions from being drawn from the data.

**Recommendation**: The authors must present the analysis performed on all valid runs (after standard quality control based on objective measures like motion, level of anesthesia or heart rate during this run, or SNR) as their primary result. The analysis based on the selected runs can, at best, be considered supplementary.

 2)  Circular Analysis in Region of Interest (ROI) Definition Similarly, the definition of the functional ROIs (pMTG, F5) from the group maps, which are then used to analyze the same data, is another form of circularity. This procedure virtually guarantees an effect but one that is biased and whose magnitude is inflated.

**Recommendation:** The standard method to avoid this bias is to use an independent localizer approach. For example, the authors could split the data for each subject in half: one half to define the ROIs, and the other, independent half to extract values and perform statistical analyses.

 3) Main contrasts

**Recommendation**: To significantly increase the impact of the current study and facilitate a direct comparison with the rich existing literature, it would be highly valuable for the authors to present the whole-brain activation maps for the 'Vocalizations > Silence' and 'Non-Vocalizations > Silence' contrasts, computed on all valid runs. Showing these fundamental activation patterns would allow the reader to visually compare the results with those from awake-fMRI studies, such as Bodin et al. (2021) and Froesel et al. (2025). This would also permit a more nuanced interpretation of the findings regarding the anterior temporal voice area (aTVA). The reported absence of aTVA selectivity is not necessarily a critical flaw; indeed, other studies on awake macaques have also reported variability in identifying this specific anterior patch. Presenting the basic activation maps would situate the current findings within this ongoing scientific discussion and would constitute a valuable contribution, independent of the outcome of the direct contrast between vocalizations and non-vocalizations.

 4) Ambiguity in the Interpretation of ROI Results The discussion of the ROI results (in F5, pMTG) is ambiguous. The authors report a difference between "vocalizations" and "non-vocalizations" but do not show whether each condition is significantly activated or deactivated relative to the baseline (silence). This makes it impossible to know if the effect is driven by activation to vocalizations, suppression by other sounds, or both.

**Recommendation**: For a correct interpretation, statistical tests to indentify whether these responses for each condition are significantly different from silence have to be provided.

 Suggestions for improvements:

 1) Transparency of Statistical Methods: The cluster correction method is incompletely described. For reproducibility, the initial voxel-level threshold used to form the clusters should be specified (also maybe the authors were too stringent with that threshold).

 2) Data Visualization: To improve the clarity of the figures, the authors could consider using inflated surfaces to better visualize activity within the sulci. The addition of anatomical atlas outlines on the activation maps is also highly recommended to help with the identification of areas.

 3) Details on Subjects and Consistency of Results: A table with the age and sex of each subject would be welcome. Showing individual activation maps (in supplementary materials) would allow an assessment of the consistency of the results across subjects.

 4) Suggestion for Exploratory Analysis: Given the presence of both males and females, the authors have a valuable opportunity to conduct an exploratory analysis of sex-based differences, a fascinating and understudied topic that would add great value to their paper. 5) Acoustic Properties of Stimuli: While the stimuli were normalized for RMS amplitude, this only accounts for overall loudness. To properly rule out low-level acoustic features as a potential confound for the "Vocalization vs. Non-Vocal" contrast, the authors should provide a more detailed acoustic analysis comparing the two categories on other key parameters (e.g., spectral content, temporal modulation, harmonic-to-noise ratio). At a minimum, a discussion of these potential uncontrolled differences is warranted.

 6) Sound Presentation Level: For the sake of reproducibility, the intensity level at which the stimuli were delivered should be explicitly reported. Please specify the sound pressure level (e.g., in dB SPL) and briefly describe how it was calibrated at the animal's head.

 7) Task Design Figure: The Methods section would be greatly clarified by the addition of a figure illustrating the task structure. A schematic depicting the sequence and timing of events within a single trial (e.g., stimulus presentation, inter-trial interval) would help readers quickly and clearly understand the experimental paradigm.

 8) Spatial Normalization Template: The Methods section should explicitly state which standard template was used for spatial normalization. The figure legend suggests it is the NMT, but this essential information needs to be clearly stated in the text for reproducibility.

 9) Spatial Smoothing and Subcortical Structures: The authors use a 3 mm smoothing kernel. This choice is particularly critical for the analysis of small subcortical structures, as the kernel may average out and obscure genuine activations in nuclei within the thalamus, midbrain, etc. It would be valuable to know if the subcortical findings are robust to this parameter, or to discuss this potential limitation.

 In conclusion, this paper presents a potentially rich dataset, but the main conclusions regarding vocalization selectivity in areas F5 and 8a are not currently supported by a rigorous and unbiased methodology. The issues of circular analysis are critical and must be addressed. Nevertheless, I am confident that the authors can resolve these points by re-analyzing their data using standard, unbiased methods. The study's potential to clarify the limitations of imaging under anesthesia is very real. Indeed, demonstrating that even with a large cohort, widespread activity outside the lateral sulcus is not necessarily present under light anesthesia would be a fundamental methodological finding for the primate imaging community. The authors are encouraged to develop the paper further along this axis, as this provides crucial guidance for researchers designing future studies with anesthetized animals. For these reasons, I recommend Approved with Reservations

Is the study design appropriate and does the work have academic merit?

Yes

Is the work clearly and accurately presented and does it cite the current literature?

Yes

If applicable, is the statistical analysis and its interpretation appropriate?

Partly

Are all the source data underlying the results available to ensure full reproducibility?

Yes

Are the conclusions drawn adequately supported by the results?

Partly

Are sufficient details of methods and analysis provided to allow replication by others?

Partly

Reviewer Expertise:

fMRI, NHP, sensory perception

I confirm that I have read this submission and believe that I have an appropriate level of expertise to confirm that it is of an acceptable scientific standard, however I have significant reservations, as outlined above.
